# Machine learning versus conventional methods for prehospital detection of stroke due to large vessel occlusion or intracranial haemorrhage

**DOI:** 10.1093/esj/aakag112

**Published:** 2026-09-18

**Authors:** Bjarty L Garcia, Luuk Dekker, Rene Bekker, Marieke J H Wermer, Ellis S van Etten, Erik W van Zwet, Jan Bosch, Adriaan C G M van Es, Ido R van den Wijngaard, Loet M H Kloos, Leo A M Aerden, Els L L M de Schryver, Henk Kerkhoff, Diederik W J Dippel, Jasper D Daems, Kirsten R I S Dorresteijn, Niels H Chavannes, Bob Roozenbeek, Nyika D Kruyt, for the LPSS and PRESTO investigators

**Affiliations:** Department of Public Health and Primary Care, Leiden University Medical Center (LUMC), Leiden, The Netherlands; Department of Mathematics, Vrije Universiteit Amsterdam, Amsterdam, The Netherlands; National eHealth Living Lab, Leiden University Medical Center (LUMC), Leiden, The Netherlands; Department of Neurology, Haga Hospital, The Hague, The Netherlands; Department of Neurology, Leiden University Medical Center (LUMC), Leiden, The Netherlands; Department of Mathematics, Vrije Universiteit Amsterdam, Amsterdam, The Netherlands; Department of Neurology, Leiden University Medical Center (LUMC), Leiden, The Netherlands; Department of Neurology, University Medical Center Groningen (UMCG), Groningen, The Netherlands; Department of Neurology, Leiden University Medical Center (LUMC), Leiden, The Netherlands; Department of Biomedical Data Sciences, Leiden University Medical Center (LUMC), Leiden, The Netherlands; Department of Research and Development, Regional Ambulance Services Hollands Midden (RAVHM), Leiden, The Netherlands; Department of Radiology, Leiden University Medical Center (LUMC), Leiden, The Netherlands; Department of Neurology, Haaglanden Medical Center, The Hague, The Netherlands; Department of Neurology, Haaglanden Medical Center, The Hague, The Netherlands; Department of Neurology, Groene Hart Ziekenhuis, Gouda, The Netherlands; Department of Neurology, Reinier de Graaf Gasthuis, Delft, The Netherlands; Department of Neurology, Alrijne Ziekenhuis, Leiderdorp, The Netherlands; Department of Neurology, Albert Schweitzer Ziekenhuis, Dordrecht, The Netherlands; Department of Neurology, Erasmus MC University Medical Center, Rotterdam, The Netherlands; Department of Neurology, Erasmus MC University Medical Center, Rotterdam, The Netherlands; Department of Public Health, Erasmus MC University Medical Center, Rotterdam, The Netherlands; Department of Neurology, Franciscus Gasthuis & Vlietland, Rotterdam, The Netherlands; Department of Public Health and Primary Care, Leiden University Medical Center (LUMC), Leiden, The Netherlands; National eHealth Living Lab, Leiden University Medical Center (LUMC), Leiden, The Netherlands; Department of Neurology, Erasmus MC University Medical Center, Rotterdam, The Netherlands; Department of Neurology, Leiden University Medical Center (LUMC), Leiden, The Netherlands; University Neurovascular Center (UNVC) Leiden-Den Haag, Leiden University Medical Center (LUMC), Leiden, The Netherlands

**Keywords:** emergency medical services, external validation, ICH, LVO, machine learning, prehospital triage, stroke scales, XGBoost

## Abstract

**Introduction:**

Adequate prehospital triage of anterior-circulation LVO (aLVO) or ICH enables direct allocation to appropriate stroke centres. Traditional triage based on clinical scales or logistic regression may miss complex predictor interactions, whereas machine learning approaches may improve diagnostic accuracy. We compared 3 prehospital triage methods to detect a composite outcome of aLVO or ICH using internal-external cross-validation.

**Patients and methods:**

We analysed emergency medical services-activated stroke codes (2018-2019) from the Leiden Prehospital Stroke Study and the Prehospital Triage of Patients With Suspected Stroke Study. We compared XGBoost (supervised machine learning [sML]), conventional logistic regression (cLR) and the Rapid Arterial oCclusion Evaluation (RACE) scale using 2-fold internal-external cross-validation. Two variable sets were evaluated: (1) routine prehospital variables (demographics, symptom duration, vital signs, glucose, neurological deficits) and (2) an extended set including medical history, anticoagulant use and point-of-care international normalised ratio. Discrimination and calibration were assessed using balanced accuracy, sensitivity, specificity, Brier scores and calibration plots.

**Results:**

Among 3320 patients, 538 (16%) had aLVO or ICH. Using routine variables, mean balanced accuracy was 82.1% for sML, 79.0% for cLR and 74.5% for RACE (*P* < .01). With extended variables, performance improved to 84.4% for sML and 80.7% for cLR (*P* < .01). The sML model achieved the highest sensitivity (76.7%) and specificity (92.1%). Calibration was good for both models, although Brier scores were lower for cLR.

**Conclusion:**

XGBoost showed modestly higher discriminative performance than cLR or RACE, while cLR showed better calibration. These findings suggest that machine learning may improve prehospital classification in selected settings, but the absolute gains were limited and require further external validation and prospective implementation studies.

## Graphical abstract

**Graphical Abstract:**
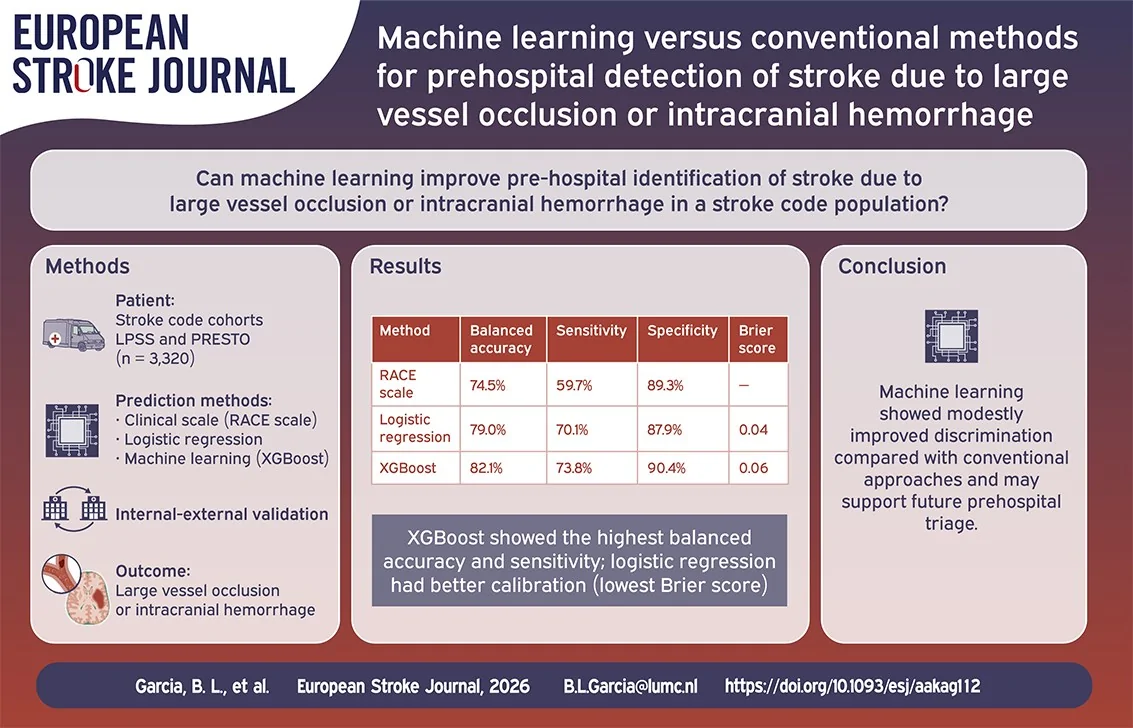

## Introduction

A reliable prehospital diagnosis of anterior-circulation LVO (aLVO) stroke or ICH can enable direct transfer to comprehensive stroke centres, thereby expediting treatment and potentially improving patient outcomes.^1^^,^^2^ For ischaemic stroke, intravenous thrombolysis (IVT) and EVT are particularly time sensitive. Similarly, for non-traumatic ICH, treatment efficacy is increasingly recognised as time-dependent—including intensive blood pressure management, reversal of coagulopathy and neurosurgical procedures.^3–5^ The prehospital decision is fundamentally a triage decision rather than a diagnostic one. Emergency medical service (EMS) professionals must determine whether a patient is likely to require comprehensive stroke-centre resources, before neuroimaging is available. Rapid access to comprehensive stroke-centre expertise is required especially for patients with aLVO. However, in light of the emerging (neurosurgical) time-sensitive treatment options, this is also the case for patients with ICH.^6^

Several prehospital aLVO stroke detection scales have been developed to identify aLVO,^7–9^ and some have also shown utility in detecting ICH.^7^ However, these scales primarily rely on neurological deficits, while incorporating additional variables routinely available in the ambulance—such as demographic characteristics or vital parameters—may improve accuracy. Moreover, advances in digital data sharing and point-of-care testing are making it increasingly feasible to include additional variables like medical history, medication use and international normalised ratio (INR) in the prehospital setting, further enhancing aLVO or ICH detection. Most studies on prehospital detection of aLVO or ICH have relied on conventional regression models.^7–9^ Yet, non-linear prediction models, such as machine learning models, are better equipped to capture complex interactions. Applying these models to stroke care may offer superior performance, particularly as larger datasets become available.^10–13^

Therefore, our aim was to compare 3 methods for detecting a composite outcome of aLVO or ICH: (1) a conventional logistic regression (cLR) model; (2) a supervised machine learning (sML) model and (3) the widely used Rapid Arterial oCclusion Evaluation (RACE) scale as a reference.^14–16^ Additionally, we explored whether expanding the routine prehospital variables with variables that could become available in the future would improve performance of the cLR and sML models.

## Patients and methods

### Study population

We used individual patient data from 2 large, prospective observational cohorts: the Leiden Prehospital Stroke Study (LPSS) and the PREhospital triage of patients with suspected STrOke (PRESTO) study.^8^^,^^9^ These cohorts included adult patients (≥18 years) for whom EMS paramedics activated an acute stroke code between July 2018 and October 2019 with each cohort drawn from 2 Dutch ambulance regions. These regions serve approximately 4 million inhabitants through 10 primary stroke centres and 5 comprehensive stroke centres. A stroke code was triggered by a positive Face-Arm-Speech-Time (FAST) test or other neurological signs at the paramedic’s clinical judgement.^7^

### Variables

We defined 2 progressively comprehensive sets of variables. First, the routine set included prehospital data routinely recorded by EMS in electronic transport charts: demographics, symptom duration, vital parameters, point-of-care blood glucose level and prehospital neurological deficits which were documented for the LPSS and PRESTO studies and allowed for reconstruction of established aLVO prediction scales.^7^ The routine set comprises variables that can be used in a deployable model under current EMS conditions. Second, as an exploratory analysis of future data availability in the extended set, we added variables that were extracted from corresponding hospital electronic patient records which we considered likely to become available in the prehospital setting in the near future. These comprised premorbid mRS scores, detailed medical history and information on anticoagulant or antithrombotic medication use and point-of-care INR measurements (for a full variable list, see Tables S1 and S3).

### Outcomes

The outcome was a composite of aLVO or ICH. Anterior-circulation LVO was defined as occlusion of the internal carotid artery, M1 or M2 part of the middle cerebral artery, A1 or A2 part of the anterior cerebral artery. Although some distal occlusions (eg, M2, A2) are increasingly classified as medium vessel occlusions in contemporary literature, we retained the original study definition to ensure consistency with the source cohorts and previously published analyses. Intracranial haemorrhage was defined as a primary intracerebral haemorrhage (including deep, lobar or posterior fossa haemorrhages), intraventricular haemorrhage or aneurysmal subarachnoid haemorrhage. Outcomes (aLVO or ICH) were extracted from hospital records. In the PRESTO study, an Imaging Core Laboratory re-evaluated all neuroimaging for presence of aLVO. In LPSS, aLVO was determined based on local assessment. Additionally, aLVO and ICH are analysed separately in the Supplement.

### Descriptive analysis

We report completeness of data, medians with IQR or means with SD for all continuous variables, as appropriate. Categorical variables are summarised using counts and percentages.

### Prediction models

The development and reporting of diagnostic models in this study adhered to the TRIPOD-AI (Transparent Reporting of a multivariable prediction model for Individual Prognosis Or Diagnosis—Artificial Intelligence) guidelines,^17^ an extension of the original TRIPOD statement tailored for studies involving machine learning. The TRIPOD-AI checklist was used to ensure comprehensive reporting of study design, data processing, model development, internal and external validation and performance assessment.

The cLR model assumes a linear relationship between the predictor variables and the log-odds of the outcome. Logistic regression remains a standard method in medical research due to its interpretability and ease of implementation for binary classification tasks.

The sML model was developed using XGBoost, a non-linear, gradient-boosted decision-tree algorithm. XGBoost combines weak learners (models) into a strong ensemble model, uses second-order Taylor approximations of the loss function and applies regularisation to reduce bias and overfitting.^11^^,^^18^^,^^19^

As a reference, we included the RACE scale, a prehospital tool for aLVO detection. The RACE scale is an ordinal scale (0–9) that assigns points based on neurological deficits (facial palsy, arm and leg motor deficits, gaze deviation and either agnosia or aphasia). In clinical practice, a score of more than 4 points is considered positive.^20^

### Model development

We performed 2-fold internal-external validation using data from the LPSS and PRESTO cohorts to assess the model’s generalisability across different geographic regions.^21^ In each fold, one cohort was used for model training, the training cohort, while the other served as the external validation set. All aspects of model development, including determining the parameters for the sML model (hyperparameter tuning), were restricted to the training cohort to avoid information leakage. Specifically, hyperparameters (eg, learning rate, tree depth) were optimised using 5-fold cross-validation within the training cohort. This cross-validation uses 80% of the training cohort for optimisation and 20% of the training cohort for validation. The model was then retrained on the training cohort using the optimal hyperparameters and subsequently evaluated on the external validation cohort, which remained completely independent throughout model development.

### Model performance

We used balanced accuracy—the mean of sensitivity and specificity—as the primary measure of discriminative performance, as it provides an unbiased assessment in the presence of class imbalance.^22^^,^^23^ Traditional accuracy metrics can be misleading when one class dominates (in this case, patients without aLVO or ICH), whereas balanced accuracy gives equal weight to both positive and negative classifications. For both the cLR and sML models, predicted probabilities were converted into binary outcomes using an optimal threshold derived from Youden’s index, which maximises the sum of sensitivity and specificity.^24^ Calibration was evaluated using Brier score and calibration curves. To quantify uncertainty, 95% CIs were estimated via 1000 bootstrap resamples of each external validation set. Balanced accuracy was statistically compared between models using McNemar’s test on the validation cohorts, which assesses disagreement in paired binary outcomes between classifiers.^25^ Model performance is summarised by the average values across both validation sets for balanced accuracy, sensitivity, specificity, positive predictive value (PPV) and negative predictive value (NPV). Finally, to illustrate the potential clinical impact of each prediction model, we constructed a hypothetical triage scenario of 1000 consecutive stroke-code patients using the observed prevalence of the composite outcome in our cohort. The numbers of correctly triaged patients, missed aLVO/ICH cases and over-triaged patients were calculated from the estimated sensitivity and specificity of each model. Uncertainty around these estimates was derived by propagating the bootstrapped 95% CIs for sensitivity and specificity obtained during the internal-external validation.

### Missing data

Missingness was assumed to be at random. To assess the plausibility of this assumption, baseline characteristics between patients with and without missing values were compared using *t*-tests and chi-squared tests. As no clinically relevant differences were observed, missing data were handled using multiple imputation by chained equations with 5 imputed datasets.^26^ All available variables were included in the imputation model. To combine results across imputations, Rubin’s rules were applied to pool the outcomes of the multivariable analyses and predictions were averaged across imputations for XGBoost.^27^ The number and proportion of missing values for each predictor and outcome are reported in Table S6, and the distribution of the number of missing predictor variables per patient is shown in Table S7.

### Software

R (version 4.2.1) was used for all statistical analyses. The “XGBoost” package (version 1.7.6) was used to implement the XGBoost model. Additional packages used included “caret” for model training and evaluation, “pROC” for receiver operating characteristic (ROC) curve analysis, “mice” for multiple imputation of missing data, “dplyr” and “tidyr” for data manipulation and “ggplot2” for data visualisation.

## Results

### Patients

In total, 3321 patients who presented under a stroke code protocol were included from the LPSS (*n* = 2007) and PRESTO (*n* = 1314) studies. One patient under the age of 18 years was excluded, resulting in a final study population of 3320 patients (Figure 1). Of these, 538 patients (16%) had the composite outcome of aLVO (295, 9%) or ICH (243, 7%). Among the remaining 2782 (83.8%) patients, 1703 (51.3%) had a non-aLVO ischaemic stroke or TIA and 1079 (32.5%) a non-stroke diagnosis (stroke mimic).

**Figure 1:**
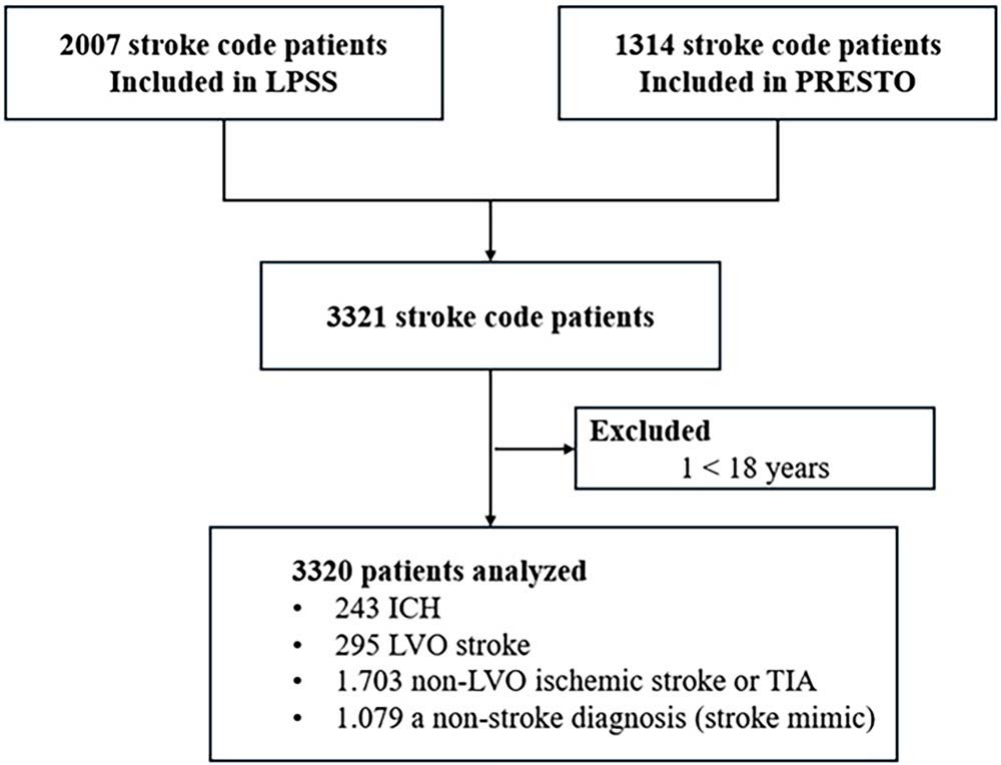
Flowchart of patient inclusion. Abbreviations: LPSS = Leiden Prehospital Stroke Study; PRESTO = Prehospital Triage of Patients With Suspected Stroke Study.

Table 1 presents baseline characteristics and data completeness for the entire cohort, as well as for patients with the composite outcome and those with other diagnoses. Compared to patients without aLVO or ICH, those with the composite outcome had a shorter median onset-to-ambulance-arrival time (39 vs 62 min), higher mean arterial blood pressure (115 vs 110 mmHg), were more likely to have atrial fibrillation (21% vs 14%) and less frequently had hypercholesterolaemia (47% vs 52%) (Table 1). The comparison of baseline characteristics between patients with and without missing variables did not yield statistically significant differences (Table S2).

**Table 1:** Prehospital routine and extended variable sets.

Prehospital variable	Total (*n* = 3320)	LVO or ICH (*n* = 538)	Other ^a^ (*n* = 2782)
**Routine variable set^b^**			
**Age, median (IQR)**	73 (62–82)	74 (64–82)	73 (61–82)
**Female sex, *n* (%)**	1607/3320 (48)	243/538 (45)	1364/2782 (49)
**Last seen well ≤ 6 h, *n* (%)**	2358/2977 (79)	405/506 (80)	1953/2471 (79)
**Mean arterial pressure, median (IQR), mmHg**	111 (97–125)	115 (101–131)	111 (97–124)
**Point of care glucose, mmol/L, median (IQR)**	6.6 (5.7–8.1)	7.1 (6.0–8.7)	6.5 (5.6–8.0)
**Prehospital neurological deficits, % (*n*/*N*)^c^**			
**Incorrectly answered questions**	26% (837/3204)	45% (231/509)	22% (606/2695)
**Abnormal speech**	42% (1356/3199)	76% (384/508)	54% (1459/2691)
**Commands** **- Both commands correctly followed**	78% (2486/3200)	55% (286/516)	82% (2200/2684)
**- 1 command correctly followed**	9% (280/3200)	16% (84/516)	7% (196/2684)
**- 0 command correctly followed**	14% (434/3200)	28% (146/516)	11% (288/2684)
**Partial or forced gaze deviation**	17% (545/3178)	47% (241/513)	11% (304/2665)
**Face drooping** **- Normal**	61% (1983/3229)	34% (181/525)	67% (1802/2704)
**- Mild facial palsy**	27% (887/3229)	34% (179/525)	26% (708/2704)
**- Severe facial palsy**	11% (359/3229)	31% (165/525)	7% (194/2704)
**Grip strength** **- Normal**	59% (1783/3018)	24% (110/465)	66% (1673/2553)
**- Decreased grip strength**	28% (843/3018)	31% (144/465)	27% (699/2553)
**- No grip strength**	13% (392/3018)	45% (211/465)	7% (181/2553)
**Arm drift** **- Normal or drift**	64% (1855/2898)	26% (114/445)	71% (1741/2453)
**- Some effort against gravity**	21% (597/2898)	23% (103/445)	20% (494/2453)
**- No effort against gravity**	15% (446/2898)	51% (228/445)	9% (218/2453)
**Leg drift** **- Normal or drift**	64% (1685/2630)	27% (113/416)	71% (1572/2214)
**- Some effort against gravity**	21% (544/2630)	25% (102/416)	20% (442/2214)
**- No effort against gravity**	15% (401/2630)	48% (201/416)	9% (200/2214)
**Agnosia** **- Recognises arm and impairment**	81% (1638/2027)	56% (159/286)	85% (1479/1741)
**- Doesn’t recognise arm OR impairment**	13% (270/2027)	20% (58/286)	12% (212/1741)
**- Doesn’t recognise both arm AND impairment**	6% (119/2027)	24% (69/286)	3% (50/1741)
**Extended variable set^d^**			
**Medical history of, *n*/*N***			
**Atrial fibrillation^e^**	15% (505/3298)	21% (114/532)	14% (391/2766)
**Diabetes mellitus**	21% (687/3299)	18% (95/533)	21% (592/2766)
**Hypertension**	62% (2032/3302)	64% (339/533)	61% (1693/2769)
**Hypercholesterolaemia**	53% (1750/3296)	47% (251/532)	54% (1499/2764)
**Primary intracerebral haemorrhage**	3% (101/3311)	4% (20/533)	3% (81/2778)
**Myocardial infarction**	11% (372/3291)	11% (61/533)	11% (311/2758)
**Peripheral artery disease**	4% (126/3243)	5% (26/529)	4% (100/2714)
**Use of antithrombotics:** **- Platelet inhibitors** **- Anti-coagulation (vitamin K antagonist or DOAC)**	36% (1190/3288) 9% (290/3274)	31% (167/531) 11% (61/531)	37% (1023/2757) 8% (229/2743)
**International normalised ratio, median (IQR)**	1.0 (0.9–1.1)	1.1 (1.0–1.3)	1.0 (0.9–1.1)
**Premorbid mRS, median (IQR)**	1 (1–3)	1 (1–2)	1 (1–3)

Abbreviations: DOAC=direct oral anticoagulant; EMS=emergency medical services; LPSS=Leiden Prehospital Stroke Scale; PRESTO=Prehospital Triage of Patients with Suspected Stroke; TIA=transient ischaemic attack.

a Non-LVO ischaemic stroke, TIA and stroke mimic.

b Prehospital variables routinely recorded by emergency medical services.

c Neurological deficits were assessed by EMS personnel in the ambulance for the LPSS and PRESTO studies.

d As routine variable set plus variables extracted from corresponding hospital electronic patient records.

e The difference between both groups is significant.

### Model performance

#### Discrimination

Using the routine variable set, sML showed higher mean balanced accuracy at 82.1% than cLR (79.0%; McNemar’s χ^2^(1) = 6.9, *P* = .008) and RACE (74.5%; χ^2^(1) = 16.4, *P* = .003) (Table 2).

**Table 2:** Mean (range of 95% CIs) performance metrics of the prediction models across both cohorts with the routine- and extended variable sets.

Variable set	Model	Balanced accuracy (%)	Sensitivity (%)	Specificity (%)	PPV (%)	NPV (%)	Brier score ^5^
**RACE^a^ items**	RACE scale	74.5 (71.3–75.8)	59.7 (55.3–61.4)	89.3 (86.8–90.5)	51.7 (47.0–53.4)	91.9 (89.2–92.8)	–
**Routine^b^**	cLR sML	79.0^1^ (77.4–80.8) 82.1^2^ (80.5–84.2)	70.1 (67.6–72.6) 73.8 (72.0–75.4)	87.9 (86.5–89.3) 90.4 (86.5–89.6)	52.8 (50.4–55.3) 59.8 (53.1–59.2)	93.8 (92.8–95.2) 94.6 (93.7–95.5)	0.04 (0.04–0.04) 0.06 (0.06–0.06)
**Extended^c^**	cLR sML	80.7^3^ (78.9–82.8) 84.4^4^ (82.2–86.0)	72.1 (69.5–74.7) 76.7 (74.6–78.4)	89.3 (88.7–90.5) 92.0 (90.5–93.8)	56.6 (55.0–58.4) 65.2 (62.5–67.9)	94.3 (93.6–95.6) 95.1 (93.9–95.8)	0.04 (0.04–0.04) 0.06 (0.06–0.06)

Abbreviations: cLR=conventional logistic regression; NPV=negative predictive value; PPV=positive predictive value; RACE=Rapid Arterial oCclusion Evaluation; sML=supervised machine learning.

1. McNemar’s test with routine variable set for LR vs RACE: *P* = .009.

2. McNemar’s test with routine variable set for ML vs LR: *P* = .008; and for ML vs RACE: *P* = .003.

3. McNemar’s test with extended variable set for cLR vs routine variable set for cLR: *P* = .043.

4. McNemar’s test with extended variable set for sML vs routine variable set for sML: *P* = .031.

5. Brier score method is given in the Supplementary material.

a Neurological deficits assessed by emergency medical services.

b Prehospital variables routinely recorded by emergency medical services.

c As routine variable set plus variables extracted from corresponding hospital electronic patient records.

Extending the variable set improved the performance for both models. Balanced accuracy of the sML increased from 82.1% to 84.4% (McNemar’s χ^2^(1) = 4.1, *P* = .031), while the balanced accuracy of the cLR increased from 79.0% to 80.7% (McNemar’s χ^2^(1) = 4.6, *P* = .043). With the extended variable set sML maintained a significant performance advantage over both cLR (χ^2^(1) = 9.4, *P* = .002) and RACE scale (χ^2^(1) = 18.2, *P* < .001).

With the routine variable set, sensitivity varied from 73.8% for sML to 70.1% for cLR and 59.7% for RACE, while specificity ranged from 90.4% for sML to 87.9% for cLR and 89.3% for RACE. Expanding the variable set improved these performance metrics of both models. For sML, sensitivity increased to 76.7% and specificity to 92.1%, while for cLR, sensitivity increased to 72.1% and specificity to 89.3%.

Table 3 summarises model performance across both cohorts. The sML model showed higher discrimination than cLR and RACE, whereas cLR showed lower Brier scores. Performance improved further when using the extended predictor set. The RACE scale showed the lowest sensitivity and overall performance across both cohorts.

**Table 3:** Performance metrics of the prediction models with the routine- and extended variable sets for each of the internal-external validation with bootstrap 95% CIs. Including sensitivity, specificity, positive predictive value (PPV), negative predictive value (NPV) and Brier score.

Variable set	Model	Validation cohort	Balanced accuracy (%)	Sensitivity (%)	Specificity (%)	PPV (%)	NPV (%)	Brier score
**RACE items^a^**	RACE Scale	PRESTO	74.5 (73.5–75.8)	59.7 (58.0–61.4)	89.3 (88.1–90.5)	51.7 (50.1–53.4)	91.9 (90.7–92.8)	–
**RACE items^a^**	RACE Scale	LPSS	72.8 (71.3–74.2)	57.4 (55.3–59.5)	88.2 (86.8–89.6)	48.9 (47.0–51.0)	90.8 (89.2–92.1)	–
**Routine^b^**	cLR	PRESTO	78.1 (77.4–79.3)	68.2 (67.6–69.0)	88.1 (87.6–89.3)	51.2 (50.4–51.7)	93.4 (92.8–94.2)	0.04 (0.04–0.04)
**Routine^b^**	cLR	LPSS	79.9 (78.9–80.8)	72.0 (71.3–72.6)	87.7 (86.5–88.7)	54.3 (53.1–55.3)	94.2 (93.7–95.2)	0.04 (0.04–0.04)
**Routine^b^**	sML	PRESTO	81.0 (80.5–81.5)	72.9 (72.0–73.4)	89.1 (88.5–89.6)	58.1 (56.9–59.2)	94.3 (93.8–95.5)	0.06 (0.06–0.06)
**Routine^b^**	sML	LPSS	83.1 (82.7–84.2)	74.7 (74.1–75.4)	91.6 (90.7–92.4)	61.5 (60.6–62.6)	94.9 (93.9–95.5)	0.06 (0.04–0.04)
**Extended^c^**	cLR	PRESTO	79.8 (78.9–80.8)	70.3 (69.5–71.3)	89.2 (88.7–90.0)	55.5 (55.0–56.1)	94.0 (93.6–95.1)	0.04 (0.04–0.04)
**Extended^c^**	cLR	LPSS	81.6 (81.2–82.8)	73.9 (73.3–74.7)	89.4 (89.0–90.5)	57.7 (57.3–58.4)	94.6 (93.6–95.6)	0.04 (0.04–0.04)
**Extended^c^**	sML	PRESTO	83.3 (82.2–83.9)	75.5 (74.6–75.9)	91.1 (90.5–92.0)	63.2 (62.5–63.8)	94.9 (93.9–95.4)	0.06 (0.06–0.06)
**Extended^c^**	sML	LPSS	85.5 (85.0–86.0)	77.9 (77.0–78.4)	93.0 (92.4–93.8)	67.2 (66.1–67.9)	95.3 (94.6–95.8)	0.06 (0.06–0.06)

Abbreviations: cLR=conventional logistic regression; LPSS=Local Prehospital Stroke Scale; NPV=negative predictive value; PPV=positive predictive value; PRESTO=Prehospital Triage for Patients with Suspected Large Vessel Occlusion; RACE=Rapid Arterial oCclusion Evaluation; sML=supervised machine learning.

a Neurological deficits assessed by emergency medical services.

b Prehospital variables routinely recorded by emergency medical services.

c As routine variable set plus variables extracted from corresponding hospital electronic patient records.

In secondary exploratory analyses using the same internal-external validation approach and the routine variable set, separate XGBoost models showed a balanced accuracy of 83.2% for aLVO-only detection and 78.5% for ICH-only detection (Tables S4 and S5). SHapley Additive exPlanations (SHAP) summary plots for the composite, aLVO-only and ICH-only models are provided in Figures S2–S4.

#### Calibration

The Brier score was 0.061–0.063 for sML and 0.041–0.044 for cLR. Brier scores were lowest for cLR compared to sML, where lower is better. The Brier scores can also be calculated compared to prevalence. With a composite outcome prevalence of 16%, the reference Brier score was 0.136, resulting in scaled Brier scores between 54% (sML) and 70% (cLR), where higher is better.^28^ The Brier scores and calibration plots during internal-external validation indicate good agreement (calibration) between predicted and observed probabilities for sML and cLR.

#### Clinical impact

Table 4 illustrates the potential impact of each model in a hypothetical triage scenario involving 1000 stroke code patients, assuming 162 true aLVO/ICH cases (as observed in our cohort) using the routine variable set. The RACE scale would miss 65 aLVO/ICH cases, cLR 48 and sML only 42. Over-triage was 90 patients with RACE, 101 with cLR, and 80 with sML. Overall, sML resulted in the highest number of correct triage decisions, correctly triaging 878 patients compared with 845 for RACE and 851 for cLR, with uncertainty shown in Table 4.

**Table 4:** Hypothetical triage scenario for 1000 stroke-code patients, including 162 with the aLVO/ICH outcome, based on average model performance across both validation cohorts using the routine variable set.

Model	Correctly triaged aLVO/ICH to CSC	Missed aLVO/ICH	Correctly triaged non-aLVO/ICH to PSC	Over-triaged non-aLVO/ICH to CSC	Total correct triage decisions
**RACE scale**	97 (90–99)	65 (63–72)	748 (727–758)	90 (80–111)	845 (817–857)
**Conventional LR**	114 (110–118)	48 (44–52)	737 (725–748)	101 (90–113)	851 (835–866)
**Supervised ML**	120 (117–122)	42 (40–45)	758 (751–768)	80(70–87)	878 (868–890)

Abbreviations: CSC=comprehensive stroke centre; LR=logistic regression; ML=machine learning; PSC=primary stroke centre; RACE=Rapid Arterial oCclusion Evaluation.

## Discussion

In this large, prospective cohort of 3320 stroke code patients with routinely available EMS data, sML with XGBoost showed modestly higher discrimination compared to conventional cLR and the RACE scale in detecting a composite outcome of aLVO or ICH, while cLR showed higher calibration. We demonstrated that compared to the other models sML provides the highest number of correctly triaged patients and the smallest numbers in both missed aLVO/ICH cases and over-triaged patients, offering the most favourable trade-off between sensitivity and specificity. Although these improvements appear modest, our hypothetical example of 162 aLVO/ICH patients underscores how modest percentage improvements can yield meaningful patient-level benefits at scale, both by reducing missed aLVO/ICH cases by 23 and avoiding unnecessary transfers for 10 stroke codes to comprehensive stroke centres (for 1000 stroke code patients: RACE missed 65 vs sML missed 42, which is a reduction of 23; over-triage 90 in RACE vs 80 in sML).

Previous studies also explored machine learning using methodology similar to ours to improve prehospital detection, mainly of patients with aLVO.^29–31^ Results of these studies varied in reported accuracy with sensitivity between 0.64 and 0.79 and specificity between 0.80 and 0.89, which is similar for sensitivity and slightly lower for specificity compared to our model and showed potential for improvement of aLVO detection in prehospital setting. Importantly, all these studies used data that were retrospectively extracted from hospital records based on assessments from neurology residents. Our study is the first to use variables that were collected by EMS paramedics in the field. This is important as it better reflects routine care since prehospital assessment, especially of neglect, speech and gaze deviation, can differ from in-hospital assessments.^32^ Moreover, prior studies used aLVO as the sole outcome, overlooking the fact that accurate allocation of patients with ICH is becoming increasingly important due to treatment being increasingly recognised as time-dependent.

Key strengths of this study include the large, prospective, well-characterised sample, the use of prehospital data acquired by EMS paramedics and the inclusion of a broad spectrum of stroke-code patients across several EMS regions (see Tables S1 and S3). This, combined with the internal-external cross-validation as well as favourable calibration metrics, supports the generalisability of our finding that sML techniques can improve detection of stroke aLVO and ICH in prehospital settings. Additionally, the study incorporates both prehospital variables that are currently routinely available, as well as variables that will likely become available in the prehospital setting with the advancement of digital data sharing—such as medical history and medication use—allowing for the evaluation of the diagnostic models under both current and future clinical conditions. Although TRIPOD-AI refers to “prediction models,” our study focuses on diagnostic classification rather than prognostic forecasting. The models are therefore best conceptualised as diagnostic models, aiming to identify patients with an ongoing LVO or intracranial haemorrhage in the prehospital setting. Another strength is the direct comparison of conventional statistical methods (cLR), advanced sML models (XGBoost) and a widely used clinical triage scale (RACE) within the same patient cohort. This comprehensive benchmarking, while following the recently published TRIPOD-AI guidelines for our models offers a reference point for future research and potential implementation in clinical practice.

Our study also has limitations. First, performance of diagnostic models is always contingent on the quality and completeness of data collected. However, our data were assembled from real-world data acquired from 2 large multi-regional cohorts, reflecting daily practice. Missingness varied across predictors, but patients with and without missing predictor data had similar baseline characteristics, supporting the plausibility of the missing-at-random assumption. Second, the feasibility of model deployment in clinical practice may decrease as more input variables are required. Still, all variables used were routinely documented in ambulance records (routine variable set) or available from in-hospital records (extended variable set) which are already part of standard care. The extended variable set was not intended to represent a model that can currently be implemented in all EMS settings. Rather, it was included to estimate the potential incremental value of future prehospital access to electronic health-record data and point-of-care testing. Furthermore, this extended variable set depends on access to patient records, prior medical history, medication use and point-of-care INR, which may not be available for unidentified patients, or patients with incomplete electronic health-record linkage. Third, although SHAP analyses improve model explainability, XGBoost remains less inherently interpretable than cLR, which may present a barrier to clinical adoption in settings where transparent decision-making is required. Fourth, context-specific features such as geographical specifics including transportation times or the level of skill of EMS paramedics could limit external validity of our findings. Therefore, further external validation in other independent EMS settings and prospective implementation studies are needed to confirm the models’ generalisability. Additionally, a possible limitation of using balanced accuracy is that it weighs false positives and false negatives equally, whereas in the prehospital setting their clinical consequences differ. To address this, we also reported sensitivity and specificity to allow readers to interpret performance in light of these differences. Fifth, although we found no clinically relevant differences between patients with and without missing predictor data, the missing-at-random assumption cannot be verified. Multiple imputation may therefore introduce or amplify bias if the probability of missingness was related to unobserved factors. Finally, although we investigated a composite outcome of aLVO or ICH, optimal prehospital triage strategies may differ between these conditions. Importantly, identifying patients at high risk of aLVO or ICH should not be interpreted as implying that identical routing is appropriate for both. Rather, the predicted probability should be integrated with local transport times, geography, stroke-network organisation and hospital capabilities to support prehospital decision-making. For example, the RACECAT secondary analysis suggested potential harm from bypass in patients with a final diagnosis of ICH in a predominantly nonurban setting with relatively long transport times, findings that may not directly generalise to more urban stroke networks.^33^ Future implementation studies should therefore evaluate class-specific prediction models for aLVO and ICH, assess their clinical and cost-effectiveness and compare model-assisted triage with neurologist-assisted ambulance triage, including teleconsultation workflows.

## Conclusion

XGBoost and logistic regression both predicted a composite aLVO or ICH outcome with high accuracy. XGBoost showed slightly higher discrimination, while logistic regression showed higher calibration. While the observed gains were numerically modest, they may translate into meaningful clinical impact at the population level, depending on local context and system capacity. Although our results are validated using internal-external cross-validation, further external validation is warranted. These findings highlight the potential of these models to improve prehospital detection of stroke patients requiring direct transfer to a comprehensive stroke centre.

## Supplementary Material

20260701_Supplement_minor_revision_aakag112

## Data Availability

The data that support the findings of this study are available from the corresponding author upon reasonable request, subject to approval by the relevant data custodians and applicable privacy regulations.
