# Supplementary material for: Machine learning versus conventional methods for prehospital detection of stroke due to large vessel occlusion or intracranial haemorrhage
Source: Eur Stroke J. 2026 Sep 18;11(9):aakag112. doi: 10.1093/esj/aakag112 (PMC13639102; doi:10.1093/esj/aakag112)
Supplement: Supplementary file 1 [file 20260701_supplement_minor_revision_aakag112.docx]

**Supplemental Material**

**Machine learning versus conventional methods for prehospital detection of stroke due to large vessel occlusion or intracranial hemorrhage**

B.L. Garcia MSc¹^,^⁴^,^¹⁸, L. Dekker MD^2,3^, R. Bekker PhD^4^, Prof. Dr. M.J.H. Wermer^3,5^, E.S. van Etten MD^3^, Dr. E.W. van Zwet^7^, J. Bosch^8^, Dr. A.C.G.M. van Es¹⁰, Dr. I.R. van den Wijngaard^10^, Dr. L.M.H. Kloos^11^, Dr. L.A.M. Aerden^12^, Dr. E.L.L.M. de Schryver^13^, Dr. H. Kerkhoff^14^, Prof. Dr. D.W.J. Dippel^15^, J.D. Daems MD^15,16^, Dr. K.R.I.S. Dorresteijn^17^, Prof. Dr. N.H. Chavannes¹^,^¹⁸, Prof. Dr. B. Roozenbeek^15^, Dr. N.D. Kruyt MD^3,6^, for the LPSS and PRESTO investigators

**CONTENTS**

**eTable 1a-c. Definitions of variables selected for the prediction model**

**eTable 2. Feature importance (XGBoost) and logistic regression coefficients for each prehospital predictor**

**eTable 3. Prehospital routine and extended variable sets for subpopulations LVO and ICH.**

**eTable 4. Mean (Range of 95% Confidence intervals) performance metrics of the prediction models for only intracranial hemorrhage (ICH) across both cohorts with the routine variable set.**

**eTable 5. Mean (Range of 95% Confidence intervals) performance metrics of the prediction models for only anterior-circulation large-vessel occlusion (aLVO) across both cohorts with the routine variable set.**

**eTable 6. Missingness of variables. Number and percentage of missing observations for prehospital and the extended variable sets. The primary outcome (aLVO/ICH) was available for all included patients. Missing percentages are calculated using the total study population (n = 3320).**

**eTable 7. Distribution of Missing Predictor Variables per Patient**

**eFigure 1. Calibration plots of sML and cLR models for prehospital aLVO/ICH detection (average calibration across predicted probabilities)**

**eFigure 2. SHAP-plot for the primary composite outcome of ICH + LVO**

**eFigure 3. SHAP-plot for the outcome of ICH**

**eFigure 4. SHAP-plot for the outcome of LVO**

**eMethods 1. Completed TRIPOD+AI Checklist**

**eMethods 2. Code of XGboost training and evaluation method**

**eMethods 3. Method for calculation of Brier score**

**eMethods 4. Bootstrapping resampling procedure**

**Supplemental Material References**

**eTable 1a-c. Definitions of variables selected for the prediction model**

a. **Demographic characteristics**

| Predictor | Type / Unit | Summary Measure | Source |
| --- | --- | --- | --- |
| Age | Years | Median (IQR) | Ambulance personnel |
| Female sex | Binary | Number (%) | Ambulance personnel |
| Time since last seen well within 6 hours | Binary | Number (%) | Ambulance personnel |
| Mean arterial pressure | mmHg | Median (IQR) | Ambulance personnel |
| Glucose | mmol/L | Median (IQR) | Ambulance personnel |

b. **Prehospital neurological deficits**

| Predictor | Type / Unit | Summary Measure | Source |
| --- | --- | --- | --- |
| Incorrectly answered questions | Binary | Number (%) | Ambulance personnel |
| Abnormal speech | Binary | Number (%) | Ambulance personnel |
| Commands followed | Categorical (0, 1, or 2) | Number (%) | Ambulance personnel |
| Partial or forced gaze deviation | Binary | Number (%) | Ambulance personnel |
| Face drooping | Categorical (normal/mild/severe) | Number (%) | Ambulance personnel |
| Grip strength | Categorical (normal/decreased/none) | Number (%) | Ambulance personnel |
| Arm drift | Categorical (normal/drift/some effort/no effort) | Number (%) | Ambulance personnel |
| Leg drift | Categorical (normal or drift/some effort/no effort) | Number (%) | Ambulance personnel |
| Agnosia | Categorical (3 levels) | Number (%) | Ambulance personnel |

c. **Predictors likely available in near future (extended set)**

| Predictor | Type / Unit | Summary Measure | Source |
| --- | --- | --- | --- |
| Atrial fibrillation | Binary | Number (%) | Hospital records |
| Diabetes mellitus | Binary | Number (%) | Hospital records |
| Hypertension | Binary | Number (%) | Hospital records |
| Hypercholesterolemia | Binary | Number (%) | Hospital records |
| Primary intracerebral hemorrhage | Binary | Number (%) | Hospital records |
| Myocardial infarction | Binary | Number (%) | Hospital records |
| Peripheral artery disease | Binary | Number (%) | Hospital records |
| Use of anti-thrombotics | Binary | Number (%) | Hospital records |
| Use of anticoagulants (VKA or DOAC) | Binary | Number (%) | Hospital records |
| International Normalized Ratio (INR) | Continuous | Median (IQR) | Hospital records |

**eTable 2. Feature importance (XGBoost) and logistic regression coefficients for each prehospital variable**

| Prehospital Variable | XGBoost Importance (%) | Logistic Regression Coefficient (β) |
| --- | --- | --- |
| Age (years) | 12.3% | 0.018 |
| Female sex | 1.7% | -0.11 |
| Last seen well ≤6h | 6.8% | 0.29 |
| Mean arterial pressure (MAP) | 4.6% | 0.012 |
| Glucose (mmol/L) | 2.1% | 0.07 |
| Incorrectly answered questions | 5.9% | 0.33 |
| Abnormal speech | 8.7% | 0.41 |
| Commands: 0 followed | 9.1% | 0.52 |
| Commands: 1 followed | 2.3% | 0.24 |
| Partial or forced gaze deviation | 10.2% | 0.61 |
| Severe facial palsy | 6.3% | 0.45 |
| No grip strength | 5.1% | 0.48 |
| Arm drift: No effort | 3.9% | 0.36 |
| Leg drift: No effort | 3.3% | 0.27 |
| Doesn't recognize arm or impairment | 1.9% | 0.14 |
| Doesn't recognize both arm and impairment | 2.5% | 0.18 |
| History of atrial fibrillation | 4.0% | 0.31 |
| History of hypertension | 3.6% | 0.22 |
| History of diabetes mellitus | 2.8% | 0.19 |
| Use of anti-thrombotics (any) | 2.0% | -0.09 |
| Premorbid mRS score | 2.7% | 0.16 |

**eTable 3.** **Prehospital routine and extended variable sets for subpopulations LVO and ICH.**

| **Pre-hospital variable** | **ICH (n=243)** | **LVO (n=295)** | **LVO or ICH (n=538)** |
| --- | --- | --- | --- |
| **Routine variable set^#^** |  |  |  |
| Age, median (IQR) | 75(64-83) | 73(64-82) | 74 (64–82) |
| Female sex, n (%) | 110/243 (45) | 133/295 (45) | 243/538 (45) |
| Last seen well ≤ 6h, n (%) | 174/223 (78) | 231/283 (82) | 405/506 (80) |
| Mean arterial pressure,  Median (IQR), mmHg | 124 (108-145) | 108 (96-123) | 115 (101-131) |
| Point of care glucose,  mmol/L, median (IQR) | 7.4 (6.1-8.9) | 6.9 (5.9-8.4) | 7.1 (6.0-8.7) |
| Pre-hospital neurological  deficits, % (n/N) |  |  |  |
| Incorrectly answered  questions, | 46% (104/228) | 45% (127/281) | 45% (231/509) |
| Abnormal speech | 71% (158/224) | 80% (226/284) | 76% (384/508) |
| Commands  - Both Commands  correctly followed | 54% (125/232) | 57% (161/284) | 55% (286/516) |
| - 1 Command correctly  followed | 17% (39/232) | 16% (45/284) | 16% (84/516) |
| - 0 Commands correctly  followed | 29% (68/232) | 27% (78/284) | 28% (146/516) |
| Partial or forced gaze  deviation | 55% (127/230) | 51% (145/283) | 53% (272/513) |
| Face drooping  - Normal | 45% (106/233) | 26% (75/292) | 34% (181/525) |
| - Mild facial palsy | 27% (62/233) | 40% (117/292) | 34% (179/525) |
| - Severe facial palsy | 28% (65/233) | 34% (100/292) | 31% (165/525) |
| Grip strength  - Normal | 29% (57/200) | 20% (53/265) | 24% (110/465) |
| - Decreased grip strength | 31% (62/200) | 31% (82/265) | 31% (144/465) |
| - No grip strength | 41% (81/200) | 49% (130/265) | 45% (211/465) |
| Arm drift  - Normal or Drift | 28% (55/194) | 24% (59/251) | 26% (114/445) |
| - Some effort against  gravity | 24% (47/194) | 22% (56/251) | 23% (103/445) |
| - No effort against gravity | 47% (92/194) | 54% (136/251) | 51% (228/445) |
| Leg drift  - Normal or Drift | 29% (50/175) | 26% (63/241) | 27% (113/416) |
| - Some effort against  gravity | 26% (45/175) | 24% (57/241) | 25% (102/416) |
| - No effort against gravity | 46% (80/175) | 50% (121/241) | 48% (201/416) |
| Agnosia  - Recognizes arm and  impairment | 62% (74/120) | 51% (85/166) | 56% (159/286) |
| - Doesn't recognize arm  OR impairment | 25% (30/120) | 17% (28/166) | 20% (58/286) |
| - Doesn't recognize both  arm AND impairment | 13% (16/120) | 32% (53/166) | 24% (69/286) |
| **Extended variable set^##^** |  |  |  |
| Medical history of, % (n/N) |  |  |  |
| Atrial fibrillation*** | 22% (52/241) | 21% (62/291) | 21% (114/532) |
| Diabetes mellitus | 13% (31/241) | 22% (64/292) | 18% (95/533) |
| Hypertension | 63% (153/241) | 64% (186/292) | 64% (339/533) |
| Hypercholesterolemia | 44% (107/241) | 49% (144/291) | 47% (251/532) |
| Primary intracerebral  hemorrhage | 7% (18/241) | 1% (2/292) | 4% (20/533) |
| Myocardial infarction | 9% (21/241) | 14% (40/292) | 11% (61/533) |
| Peripheral artery disease | 4% (9/240) | 6% (17/289) | 5% (26/529) |
| Use of anti-thrombotics:  - platelet inhibitors  - anti-coagulation (vitamin  K antagonist or DOAC). | 28% (69/243) 14% (33/243) | 33% (98/295)  9% (28/295) | 31% (167/531)  11% (61/531) |
| International Normalized  Ratio, median (IQR) | 1.1 (1.0–1.3) | 1.1 (1.0–1.3) | 1.1 (1.0–1.3) |
| Premorbid modified Rankin  Scale (mRS), median (IQR) | 1 (1–2) | 1 (1–2) | 1 (1–2) |

***** non-LVO ischemic stroke, TIA and stroke mimic
** Neurological deficits were assessed by EMS personnel in the ambulance for the LPSS and PRESTO studies
*** The difference between both groups is significant

#: prehospital variables routinely recorded by emergency medical services
##: as routine variable set plus variables extracted from corresponding hospital electronic patient records

**eTable 4. Mean (Range of 95% Confidence intervals) performance metrics of the prediction models for only intracranial hemorrhage (ICH) across both cohorts with the routine variable set.**

| **Variable Set** | **Model** | **Balanced Accuracy (%)** | **Sensitivity (%)** | **Specificity (%)** | **PPV (%)** | **NPV (%)** | **Brier Score**^2^ |
| --- | --- | --- | --- | --- | --- | --- | --- |
| Routine ^#^ | cLR  sML | 75.1  (72.1-77.7)  78.5^1^  (76.5-81.2) | 88.7  (85.6-91.6)  91.2  (89.2-93.7) | 61.4  (59.3-63.5)  63.7  (61.4-66.9) | 15.4  (14.2-17.1)  16.6  (14.7-18.1) | 98.6  (97.9-99.5)  98.9  (98.1-99.6) | 0.05  (0.05-0.05)  0.06  (0.06-0.06) |

1 McNemar’s test with routine variable set for ML versus LR: p=0.007

2 Brier score method is given in Supplement

#: prehospital variables routinely recorded by emergency medical services

**eTable 5. Mean (Range of 95% Confidence intervals) performance metrics of the prediction models for only anterior-circulation large-vessel occlusion (aLVO) across both cohorts with the routine variable set.**

| **Variable Set** | **Model** | **Balanced Accuracy (%)** | **Sensitivity (%)** | **Specificity (%)** | **PPV (%)** | **NPV (%)** | **Brier Score**^2^ |
| --- | --- | --- | --- | --- | --- | --- | --- |
| Routine ^#^ | cLR  sML | 79.2  (77.5-80.9)  83.2^1^  (81.6-85.2) | 83.5  (80.2-85.1)  87.8  (85.4-89.0) | 75.0  (74.3-77.9)  79.4  (76.4-83.5) | 24.6  (22.4-26.8)  29.4  (28.5-31.1) | 97.9  (95.6-99.1)  98.5  (97.2-99.2) | 0.05  (0.05-0.05)  0.06  (0.06-0.06) |

1 McNemar’s test with routine variable set for ML versus LR: p=0.007

2 Brier score method is given in Supplement

#: prehospital variables routinely recorded by emergency medical services

**eTable 6. Missingness of variables. Number and percentage of missing observations for prehospital and the extended variable sets. The primary outcome (aLVO/ICH) was available for all included patients. Missing percentages are calculated using the total study population (n = 3320).**

| **Variable** | **Available (n)** | **Missing (n)** | **Missing (%)** |
| --- | --- | --- | --- |
| **Outcome (aLVO/ICH)** | **3320** | **0** | **0.0** |
| **Age** | **3320** | **0** | **0.0** |
| **Female sex** | **3320** | **0** | **0.0** |
| **Last seen well ≤6 h** | **2977** | **343** | **10.3** |
| **Mean arterial pressure** | **3320** | **0** | **0.0** |
| **Point-of-care glucose** | **3320** | **0** | **0.0** |
| **Incorrectly answered questions** | **3204** | **116** | **3.5** |
| **Abnormal speech** | **3199** | **121** | **3.6** |
| **Commands** | **3200** | **120** | **3.6** |
| **Partial or forced gaze deviation** | **3178** | **142** | **4.3** |
| **Face drooping** | **3229** | **91** | **2.7** |
| **Grip strength** | **3018** | **302** | **9.1** |
| **Arm drift** | **2898** | **422** | **12.7** |
| **Leg drift** | **2630** | **690** | **20.8** |
| **Agnosia** | **2027** | **1293** | **38.9** |
| **Atrial fibrillation** | **3298** | **22** | **0.7** |
| **Diabetes mellitus** | **3299** | **21** | **0.6** |
| **Hypertension** | **3302** | **18** | **0.5** |
| **Hypercholesterolemia** | **3296** | **24** | **0.7** |
| **Previous intracerebral hemorrhage** | **3311** | **9** | **0.3** |
| **Myocardial infarction** | **3291** | **29** | **0.9** |
| **Peripheral artery disease** | **3243** | **77** | **2.3** |
| **Platelet inhibitor use** | **3288** | **32** | **1.0** |
| **Anticoagulant use** | **3274** | **46** | **1.4** |

**eTable 7. Distribution of Missing Predictor Variables per Patient**

| **Number of missing predictor variables** | **Patients, n (%)** |
| --- | --- |
| **0** | **1187 (35.8)** |
| **1** | **923 (27.8)** |
| **2** | **582 (17.5)** |
| **3** | **299 (9.0)** |
| **4** | **138 (4.2)** |
| **5** | **73 (2.2)** |
| **6** | **24 (0.7)** |
| **7** | **11 (0.3)** |
| **≥8** | **83 (2.5)** |
| **Total** | **3320 (100.0)** |

**eFigure 1. Calibration plots of sML and cLR models for prehospital aLVO/ICH detection (average calibration across predicted probabilities)**

**eFigure 2. SHAP-plot for the XGBoost primary composite outcome of ICH + LVO**

**eFigure 3. SHAP-plot for the XGBoost outcome of ICH**

**eFigure 4. SHAP-plot for the XGBoost outcome of LVO**

**eMethods 1. Completed TRIPOD+AI Checklist**

**TRIPOD+AI Checklist**

Manuscript title: Machine learning versus conventional methods for prehospital detection of stroke due to large vessel occlusion or intracranial hemorrhage

Checklist completed by: B.L. Garcia, Senior Data Scientist (OLVG), PhD Candidate (LUMC)

Date: 12 June 2025 (revised 13 july 2025)

Reporting guideline followed: TRIPOD+AI statement, Collins GS, Moons KGM, Dhiman P, et al. BMJ 2024;385:e078378. doi:10.1136/bmj-2023-078378

Purpose: This checklist documents adherence to the TRIPOD-ML guidelines for transparent reporting of the development and validation of supervised machine learning prediction models in medical research.

| Item | Description | Section | Where Reported |
| --- | --- | --- | --- |
| 1 | Title | TITLE | Title page (lines 1–2) |
| 2 | Abstract | ABSTRACT | Abstract (lines 5–20) |
| 3a | Healthcare context and rationale | INTRODUCTION | Introduction (para 1–2) |
| 3b | Target population and purpose | INTRODUCTION | Introduction (para 3) |
| 3c | Health inequalities | INTRODUCTION | Introduction |
| 4 | Objectives | INTRODUCTION | Introduction (last paragraph) |
| 5a | Sources of data | METHODS | Methods > Study population (para 1) |
| 5b | Dates of participant data | METHODS | Methods > Study population (para 1) |
| 6a | Study setting | METHODS | Methods > Study population (para 1) |
| 6b | Eligibility criteria | METHODS | Methods > Study population (para 1) |
| 6c | Treatments received | METHODS | Not applicable |
| 7 | Data pre-processing | METHODS | Methods > Variables & Model development |
| 8a | Outcome definition | METHODS | Methods > Outcomes |
| 8b | Subjective outcome assessment | METHODS | Methods > Outcomes |
| 8c | Blinding of outcome assessment | METHODS | Not reported |
| 9a | Choice of initial predictors | METHODS | Methods > Variables |
| 9b | Definition of predictors | METHODS | Methods > Variables |
| 9c | Subjective predictor assessment | METHODS | Not applicable |
| 10 | Sample size justification | METHODS | Not explicitly justified; partly in 'Patients' section |
| 11 | Missing data handling | METHODS | Methods > Missing data |
| 12a | Use of data in analysis | METHODS | Methods > Model development |
| 12b | Handling of predictors | METHODS | Methods > Model development |
| 12c | Model type and steps | METHODS | Methods > Prediction models |
| 12d | Heterogeneity handling | METHODS | Methods > Outcome measures |
| 12e | Performance measures | METHODS | Methods > Model performance |
| 12f | Model updating | METHODS | Not applicable; no model updating reported |
| 12g | Model predictions for evaluation | METHODS | Methods > Model performance |
| 13 | Class imbalance | METHODS | Methods > Model performance (balanced accuracy) |
| 14 | Fairness | METHODS | Methods > Model development |
| 15 | Model output | METHODS | Methods > Prediction models |
| 16 | Training vs evaluation differences | METHODS | Methods > Model development |
| 17 | Ethical approval | METHODS |  |
| 18a | Funding | OPEN SCIENCE | Sources of Funding |
| 18b | Conflicts of interest | OPEN SCIENCE | Sources of Funding |
| 18c | Protocol | OPEN SCIENCE | Standard Protocol Approvals, Registrations,  and Patient Consents |
| 18d | Registration | OPEN SCIENCE | Not reported |
| 18e | Data sharing | OPEN SCIENCE | Not reported |
| 18f | Code sharing | OPEN SCIENCE | Supplement |
| 19 | Patient & public involvement | OPEN SCIENCE | Not reported |
| 20a | Participant flow | RESULTS | Results > Patients (first paragraph) |
| 20b | Participant characteristics | RESULTS | Results > Table 1 |
| 20c | Comparison with development data | RESULTS | Not explicitly; implied across 'Results' |
| 21 | Participants and events in analysis | RESULTS | Results > Model development (patients per cohort) |
| 22 | Full model specification | RESULTS | Full model and coefficients provide in Supplements |
| 23a | Model performance | RESULTS | Results > Model performance |
| 23b | Performance heterogeneity | RESULTS | Not reported |
| 24 | Model updating results | RESULTS | Not applicable; no updating done |
| 25 | Interpretation | DISCUSSION | Discussion (first 3 paragraphs) |
| 26 | Limitations | DISCUSSION | Discussion (4th paragraph) |
| 27a | Handling poor quality input data | DISCUSSION | Discussion (5th paragraph) |
| 27b | User interaction and expertise | DISCUSSION | Discussion (5th paragraph) |
| 27c | Next research steps | DISCUSSION | Discussion (final paragraph) |

**eMethods 2. Code of XGboost training and evaluation method**

dfTrainXGB <- dfTrain %>%

mutate(across(starts_with("ph_i_"), as.factor)) %>%

mutate(across(starts_with("bl_hist_"), as.factor))

dfTrainXGB$sex <- as.factor(dfTrainXGB$sex)

dfTrainXGB$wakeup_stroke <- as.factor(dfTrainXGB$wakeup_stroke)

dfTrainXGB$tactiele_extinctie <- as.factor(dfTrainXGB$tactiele_extinctie)

dfTrainXGB$bl_premrs.x <- as.factor(dfTrainXGB$bl_premrs.x)

dfTrainXGB$bl_premrs.y <- as.factor(dfTrainXGB$bl_premrs.y)

dfTrainXGB$ph_avpu <- as.factor(dfTrainXGB$ph_avpu)

dfTrainXGB$race_v2 <- as.factor(dfTrainXGB$race_v2)

dfTrainXGB$race_cutoff_v2 <- as.factor(dfTrainXGB$race_cutoff_v2)

dfTrainXGB <- dfTrainXGB %>%

mutate(across(starts_with("bl_med"), as.factor))

dfTestXGB <- dfTest %>%

mutate(across(starts_with("ph_i_"), as.factor)) %>%

mutate(across(starts_with("bl_hist_"), as.factor))

dfTestXGB <- dfTestXGB %>%

mutate(across(starts_with("bl_med"), as.factor))

dfTestXGB$sex <- as.factor(dfTestXGB$sex)

dfTestXGB$wakeup_stroke <- as.factor(dfTestXGB$wakeup_stroke)

dfTestXGB$tactiele_extinctie <- as.factor(dfTestXGB$tactiele_extinctie)

dfTestXGB$bl_premrs.x <- as.factor(dfTestXGB$bl_premrs.x)

dfTestXGB$bl_premrs.y <- as.factor(dfTestXGB$bl_premrs.y)

dfTestXGB$ph_avpu <- as.factor(dfTestXGB$ph_avpu)

dfTestXGB$race_v2 <- as.factor(dfTestXGB$race_v2)

dfTestXGB$race_cutoff_v2 <- as.factor(dfTestXGB$race_cutoff_v2)

ctrl <- trainControl(method = "cv", number = 5, classProbs = TRUE, summaryFunction = twoClassSummary, verboseIter = TRUE)

neg <- sum(dfTrainXGB$ICH_LVO == "no")

pos <- sum(dfTrainXGB$ICH_LVO == "yes")

class_weights <- ifelse(dfTrainXGB$ICH_LVO == "yes", neg/pos, 1)

grid <- expand.grid(

nrounds = c(50,100),

max_depth = c(3,6,9),

eta = c(0.01, 0.05, 0.1),

gamma = c(0,1,5),

colsample_bytree = c(0.7,1),

min_child_weight = c(1,3,5),

subsample = c(0.8,1)

)

xgb_model <- train(ICH_LVO ~ ., data = dfTrainXGB, method = "xgbTree", trControl = ctrl, tuneGrid = grid,

metric = "ROC", verbose = FALSE, preProcess = NULL, weights = class_weights)

xgb_pred_prob <- predict(xgb_model, newdata = dfTestXGB, type = "prob")[,2]

roc_xgb <- roc(dfTest$ICH_LVO, xgb_pred_prob)

**eMethods 3. Method for calculation of Brier score**

For binary outcomes:

$$Brier Score=\frac{1}{N}\sum_{i=1}^{N} {{(f}_{i}-o\_i)}^{2}$$

the Brier score was calculated as the mean squared difference between the predicted probability and the observed outcome. Lower values indicate better probabilistic accuracy.

Where:

- N = number of predictions
- $f_{i}=$ predicted probability of the positive class (ICH + aLVO) for observation i

$o_{i}=$ actual outcome for observation i (0 or 1)

**eMethods 4. Bootstrapping resampling procedure**

To evaluate the internal validity and robustness of our predictive model, we applied a **bootstrap resampling procedure**. Specifically, we performed **1000 bootstrap iterations** to estimate the variability and confidence intervals of key performance metrics, including the area under the receiver operating characteristic curve (AUC), Brier score, and calibration parameters (slope and intercept).

To estimate uncertainty around model performance, we performed 1,000 bootstrap resamples of each external validation cohort. Performance metrics were recalculated in each bootstrap sample, and 95% confidence intervals were derived from the empirical distribution of the bootstrap estimates. This procedure allows for the estimation of **optimism-corrected performance** and provides a measure of the model’s stability across different samples drawn from the same population.

All analyses were conducted using R 4.2.1 with the boot package.

**Supplemental Material References**

- Liu, X., et al. (2024). Transparent reporting of multivariable prediction models developed or validated using machine learning techniques: the TRIPOD-ML (AI) guidelines. *BMJ*, 385:e079808.
- TRIPOD+AI statement, Collins GS, Moons KGM, Dhiman P, et al. BMJ 2024;385:e078378. doi:10.1136/bmj-2023-078378
